# Pediatric Patients with Osteomyelitis and/or Septic Joint Undergoing Surgical Debridement Have Equivalent Short-Term Outcomes with or without Preoperative MRI

**DOI:** 10.3390/children11030300

**Published:** 2024-03-02

**Authors:** E. Graham Englert, Collin L. Braithwaite, Maria E. Aguirre-Flores, Aaron W. Lam, Mohamed Sarraj, Abigail Kumagai, E. Dimitra Bednar, Adam M. Gordon, Ibrahim Salama, Jacob Keeley, Indu Pathak, Waleed Kishta, Ahmed M. Thabet, Amr Abdelgawad, Ehab Saleh

**Affiliations:** 1 Department of Orthopaedic Surgery, Beaumont Hospital Royal Oak, Royal Oak, MI 48073, USA; ehab.saleh3@corewellhealth.org; 2 Department of Orthopedic Surgery, Oakland University William Beaumont School of Medicine, Rochester, MI 48309, USA; clbraithwaite@oakland.edu (C.L.B.); jkeeley@oakland.edu (J.K.); 3 Department of Pediatrics, Texas Tech University Health Sciences Center, El Paso, TX 79905, USA; ethel.aguirre@ttuhsc.edu (M.E.A.-F.); ipathak@northwell.edu (I.P.); 4 Department of Orthopedic Surgery, Maimonides Medical Center, Brooklyn, NY 11219, USA; aarlam@maimonidesmed.org (A.W.L.); adgordon@maimonidesmed.org (A.M.G.); aabdelgawad@maimonidesmed.org (A.A.); 5 Division of Orthopaedic Surgery, McMaster University, Hamilton, ON L8S 4L8, Canada; mohamed.sarraj@medportal.ca (M.S.); kishtaw@mcmaster.ca (W.K.); 6 Department of Ophthalmology and Visual Sciences, The Ohio State University College of Medicine, Columbus, OH 43210, USA; 7 Department of Medicine, University of Toronto, Toronto, ON M1C 1A4, Canada; dimitra.bednar@medportal.ca; 8 Paul L. Foster School of Medicine, Texas Tech University Health Sciences Center El Paso, El Paso, TX 79905, USA; isalama@ttuhsc.edu; 9 Department of Orthopaedic Surgery, Texas Tech University Health Sciences Center, El Paso, TX 79905, USA; ahmed-thabet.hagag@ttuhsc.edu

**Keywords:** MRI, septic arthritis, osteomyelitis, pediatric

## Abstract

The purpose of this study was to determine if short-term outcomes differed for pediatric patients with suspected musculoskeletal infection with or without a preoperative MRI. This was a multicenter, retrospective review of patients aged 0–16 years who presented with atraumatic extremity pain, underwent irrigation and debridement (I&D), and received at least one preoperative or postoperative MRI over a 10-year period. Primary outcomes were time to OR, total I&Ds, readmission rate, time from OR to discharge, and total number of MRIs. Secondary outcomes entailed the rate at which concurrent osteomyelitis was identified in patients with septic arthritis and the extent of the resulting surgical debridement. Of the 104 patients, 72.1% had a preoperative MRI. Patients with a preoperative MRI were significantly less likely to have surgery on the day of admission. No difference was found between groups regarding total I&Ds, readmission rate, time from OR to discharge, and total number of MRIs. Of the 57 patients diagnosed with septic arthritis, those with a preoperative MRI were significantly more likely to have concurrent osteomyelitis identified and to undergo bony debridement in addition to arthrotomy of the joint. In conclusion, patient outcomes are not adversely affected by obtaining a preoperative MRI despite the delay in time to OR. Although preoperative MRI can be beneficial in ruling out other pathologies and identifying the extent of concurrent osteomyelitis, the decision to obtain a preoperative MRI and timing of surgery should be left to the discretion of the treating surgeon.

## 1. Introduction

In the workup of pediatric patients with suspected septic arthritis or osteomyelitis, a common dilemma for orthopedic surgeons is whether to obtain a preoperative magnetic resonance imaging (MRI) study. The incidence of severe deep musculoskeletal infections is increasing in the community due to growing challenges such as Methicillin-resistant Staphylococcus aureus (MRSA) [1]. MRI is generally preferred for identifying and characterizing intraosseous and extraosseous musculoskeletal deep infections in pediatric patients due to its high sensitivity and specificity [2]. Including MRI as a standard component of an infectious workup has been suggested due to the inability of clinical exams and laboratory studies alone to differentiate between various disease processes including infection, oncological pathology, and trauma [3,4]. Many surgeons advocate for the routine use of preoperative MRI in pediatric patients with concern for septic hip due to its utility in differentiating between septic arthritis and periarticular pyomyositis [5]. Advocates for routine preoperative MRI suggest that it can play an important role in determining the extent of the causative infection and the necessary surgical debridement [6,7]. Despite its diagnostic utility, younger patients frequently require anesthesia to complete the MRI [7]. Although sedation is generally safe, there are risks ranging from short-term laryngospasm to possible long-term effects on neurocognitive development [8,9]. Furthermore, MRI cannot always be completed expediently at certain centers. Significant delays in care can lead to complications such as osteonecrosis, chondrolysis, worsening clinical deterioration, and sepsis [6,10]. Additionally, MRI is an expensive imaging modality, especially when gadolinium and/or sedation are necessary [7].

Various algorithms have been developed to assist pediatric orthopedic surgeons in evaluating patients with atraumatic extremity pain which may have an infectious etiology [10,11]. These can be traced back to the criteria described by Kocher et al. more than 20 years ago [12]. In the era of widespread MRI use in medicine, the crucial aspect of these clinical instruments often hinges on the surgeon’s clinical discretion regarding when to obtain an MRI [2]. The primary purpose of this multicenter, retrospective review is to examine how the timing of MRI impacts time to the operating room (OR) and various short-term outcomes including time from surgery to discharge, readmission rate, repeat irrigation and debridement (I&D), and total number of MRIs. The secondary aim of this study is to delineate if preoperative MRI impacts the rate in which concurrent osteomyelitis is identified in patients with a septic joint and the extent of the resulting surgical debridement.

## 2. Materials and Methods

Four tertiary pediatric referral centers in the United States and Canada agreed to participate in this retrospective review. American locations included Royal Oak (Michigan), El Paso (Texas), and New York City (New York). The Canadian location was Hamilton (Ontario). Institutional Review Board approval was obtained in each participating institution. Queries were conducted of each institution’s electronic medical records for patient encounters from January 2010 through December 2019. Patients were included if they had (I) an MRI of an extremity, (II) a diagnosis of osteomyelitis and/or septic arthritis, (III) one or more I&Ds, and (IV) a positive aspiration result (as defined by a positive Gram-stain or a synovial WBC count ≥50,000 cells per microliter) and at least one of the following: T > 38.5, ESR > 40, CRP > 2, or WBC > 12,000. The search was limited to patients 0 to 16 years of age.

Patients with multiple encounters for the same concern were combined into a single study identifier. Data from the patient’s initial presentation, including age, gender, duration of symptoms, temperature, laboratory test results, and aspirations, were recorded for each patient. Additional information obtained included the time from admission to surgery, the time from surgery to discharge, the number of debridements, instances of readmissions, and the total number of MRIs. Patients were divided into two groups, those that received a preoperative MRI and those that only had a postoperative MRI. Primary outcomes were time to OR, total I&Ds, readmission rate, time from OR to discharge, and total number of MRIs. Secondary outcomes entailed the rate at which concurrent osteomyelitis was identified in patients with septic arthritis and the extent of the resulting surgical debridement.

A subgroup analysis was then performed on patients diagnosed with septic arthritis with or without concurrent osteomyelitis. The aforementioned primary and secondary outcomes were once again investigated between patients with and without preoperative MRI. Additionally, the rate of concurrent osteomyelitis in patients within our septic arthritis subgroup was calculated for patients with and without preoperative MRI as well the rate at which the presence of osteomyelitis required additional surgical debridement. Finally, the rate of concurrent osteomyelitis and necessity for additional surgical debridement were investigated for specific joints including shoulder, elbow, wrist, hip, ankle, and knee.

Data were analyzed first overall and then stratified according to whether a patient received a preoperative MRI. Categorical variables were represented using frequencies and percentages, whereas continuous variables were displayed using medians and interquartile ranges (IQRs). To compare patients with preoperative MRIs to those without, statistical testing was performed. Categorical variables were analyzed using Chi-Square tests when appropriate, whereas continuous variables were analyzed using *t*-tests where applicable. Wilcoxon rank-sum testing was utilized as a nonparametric alternative in instances where conditions were not met. The statistical test chosen for a given variable is denoted with a Roman numeral subscript in the results tables. All testing was two-sided with an alpha of 0.05. Analyses were conducted using SAS v9.4.

## 3. Results

A total of 442 patients were identified from the initial query of patients between ages 0 and 16 who had an MRI of an extremity and a diagnosis of osteomyelitis and/or septic arthritis. A total of 338 were removed for not undergoing surgical debridement, being duplicate encounters, or not meeting our aspiration/laboratory value inclusion criteria, leaving 104 patients in the total population. As seen in Table 1, the mean age of the cohort was 7.3 years, and 38.5% of patients were female. Patients presented, on average, 7.3 days after symptoms began. In total, 72.1% of patients had a preoperative MRI. When comparing patients with and without preoperative MRI, no differences were found regarding age (*p* = 0.17), gender (*p* = 0.20), symptomatic duration (*p* = 0.58), temperature (*p* = 0.18), ESR (*p* = 0.90), CRP (*p* = 0.39), WBC (*p* = 0.31), or positive blood cultures on admission (*p* = 0.71). Patients that did not have a preoperative MRI were more likely to have preoperative joint aspiration (*p* < 0.00001); however, their aspiration results did not differ from those patients with a preoperative MRI (*p* = 0.15). Patients with a preoperative MRI were significantly less likely to have surgery on the day of admission (*p* = 0.0037).

Among the patients in our study, 45.2% were diagnosed with osteomyelitis without a septic joint, 32.7% had a septic joint without osteomyelitis, and 22.1% had septic arthritis with concurrent osteomyelitis. As seen in Table 2, when comparing patients with and without preoperative MRI, no significant differences were found regarding time from surgery to discharge (*p* = 0.57), total number of I&Ds (*p* = 0.23), readmission rate (*p* = 0.24), and total number of MRIs (*p* = 0.23).

A subgroup analysis of the 57 patients diagnosed with septic arthritis with or without concurrent osteomyelitis once again revealed no differences between patients with and without preoperative MRI regarding age (*p* = 0.72), gender (*p* = 0.46), duration of symptoms (*p* = 0.71), temperature (*p* = 0.27), ESR (*p* = 0.57), CRP (*p* = 0.85), WBC (*p* = 0.58), and positive blood culture (*p* = 0.77), as seen in Table 3.

As was observed in the larger cohort, patients who had a preoperative MRI were less likely to have undergone preoperative joint aspiration (*p* = 0.018), but their aspiration results did not significantly differ from those patients without a preoperative MRI (*p* = 0.20). As seen in Table 4, patients with a preoperative MRI were less likely to undergo surgery on the day of admission (*p* = 0.023), but there were no significant differences in the time from surgery to discharge (*p* = 0.77), total number of surgical debridements (*p* = 0.17), likelihood of readmission (*p* = 1.0), or total number of MRIs (*p* = 0.46).

**Figure 1:**
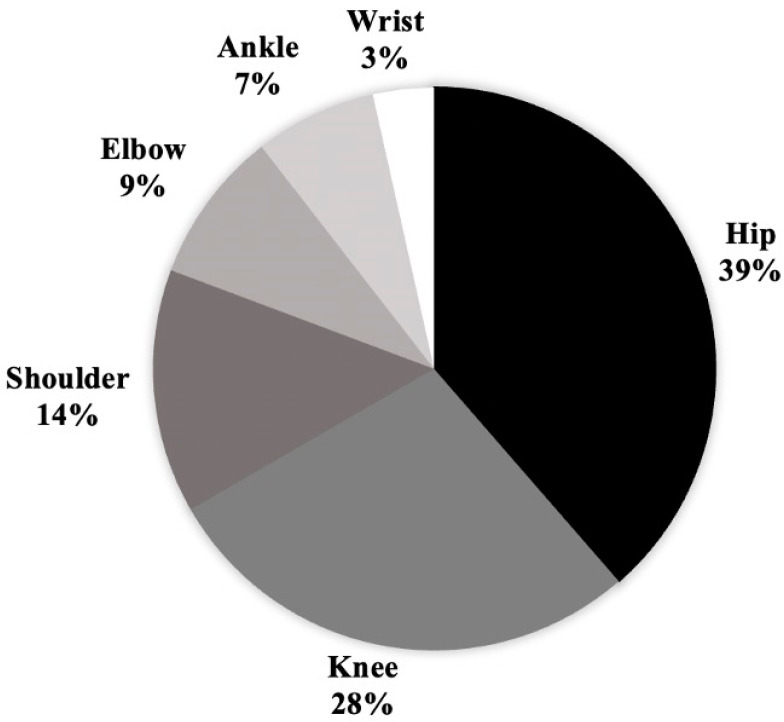
Anatomic location of pediatric patients with septic arthritis requiring debridement.

In patients with septic arthritis, the most commonly infected joint was the hip (38.6%), followed by the knee (28.1%) and shoulder (14.0%) (Figure 1).

Of the 57 patients with septic arthritis, 23 (40.4%) had concurrent osteomyelitis. Nine patients (15.8%) underwent concurrent bony debridement beyond debridement of the joint alone. As seen in Table 5, patients who had a preoperative MRI were significantly more likely to have concurrent osteomyelitis identified (*p* = 0.026) and to undergo bony debridement in addition to arthrotomy of the infected joint (*p* = 0.031). When investigating specific joints, these differences were found to be significant for the ankle for both concurrent osteomyelitis (*p* = 0.046) and bony debridement (*p* = 0.046) and approached significance for diagnosing concurrent osteomyelitis in the hip (*p* = 0.068).

## 4. Discussion

When treating pediatric patients with suspected musculoskeletal infection, providers must often balance obtaining sufficient data to be confident in a diagnosis with timely intervention. This is especially crucial when a patient is clinically deteriorating and/or there is concern for a septic joint. MRI is a powerful tool with high sensitivity and specificity for diagnosing osteomyelitis and septic arthritis [13,14]. Ultrasonography is another option to evaluate for a joint effusion and is an important tool in the workup of these patients, but MRI has higher sensitivity and specificity as well as increased interobserver reliability. MRI also proves valuable in ruling out mimickers of a septic joint such as neoplasm and transient synovitis [15,16]. A recent retrospective study of 93 patients demonstrated a reduction in unplanned returns to the operating room from 50% to less than 27% after implementing new guidelines that mandated preoperative MRIs for all pediatric patients with suspected periarticular infections [17]. The downsides to preoperative MRI include its high costs and the additional time necessary to use the technique at most hospitals, especially when anesthesia is necessary [13,14]. Even with the extensive resources present at the pediatric tertiary referral centers included in this study, patients who underwent preoperative MRI experienced significantly greater time to surgery in both the main cohort and septic joint subgroup.

The harm of delaying surgery to obtain MRI is unclear, and little has been previously published on this topic. A retrospective review of 105 pediatric patients with septic arthritis by Telleria et al. found that delayed diagnosis, as defined by a previous evaluation by a medical provider attributing the patient’s extremity pain to a benign condition, was significantly associated with multiple surgeries [18]. Other studies have found that initiating treatment within five days can avert other serious complications such as avascular necrosis (AVN) of the hip [19]. Our study revealed no differences in short-term outcomes such as reoperation rate, readmission rate, or time to discharge in patients who had preoperative MRI compared to those who did not. This held true in the septic arthritis subgroup, which likely encompassed patients most sensitive to delays in care. To our knowledge, no other study has investigated short- or long-term outcomes in patients who experienced delays lasting less than 48 h to obtain preoperative MRI.

Recent studies have highlighted the utility of preoperative MRI in determining the extent of periarticular infection in patients with septic arthritis. In 2016, Rosenfeld et al. found that 59% of patients in their retrospective cohort with septic arthritis had adjacent foci. Age greater than 3.6 years, CRP > 13.8 mg/L, duration of symptoms > 3 days, platelets < 314 × 10^3^, and ANC > 8.6 × 10^3^ cells/mL were found to be predictive of adjacent infection [20]. A multicenter, retrospective review by Murphy et al. of pediatric patients with septic hips in 2019 found a 40% rate of concurrent osteomyelitis. Adjacent bony infectious foci were associated with repeat surgical intervention with an odds ratio of 3.4 [21]. A 2013 retrospective review by Montgomery et al. of 200 patients with septic arthritis found a concurrent osteomyelitis rate of 21.5% [22]. A study by Ernat et al. in 2017 specifically investigating pediatric septic shoulders found a concurrent osteomyelitis rate of 68% based on MRI results [23].

Our study found a similarly high rate of concurrent osteomyelitis in patients with septic joints. Over 40.4% of patients had adjacent bony foci, with 15.8% requiring bony debridement beyond debridement of the joint. Patients in our study who underwent preoperative MRI were significantly more likely to be diagnosed with concurrent osteomyelitis and undergo bony debridement. However, patients who did not undergo preoperative MRI did not appear to experience worse short-term outcomes in terms of reoperation and readmission rates, as well as time to discharge. These findings are consistent with the study performed by Murphy et al. in 2019 [21]. Interestingly, as seen in Table 5, joints with intra-articular metaphysis such as the hip, elbow, and ankle had higher rates of adjacent osteomyelitis than the wrist did, which has also been demonstrated in the prior literature [14].

Limitations include the retrospective nature of the study and the difficulty of controlling for possible confounding variables. In the overall cohort, patients without preoperative MRI were significantly more likely to have had an aspiration, reflecting surgeons feeling sufficiently confident in their diagnosis to proceed to the operating room without advanced imaging. When controlling for diagnosis and limiting our analysis to patients with septic arthritis, we found that patients with preoperative MRI were more likely to be diagnosed with concurrent osteomyelitis and undergo bony debridement. Due to the absence of randomization, we cannot establish a causative relationship. However, it is possible that having the preoperative MRI provided sufficient information to diagnose concurrent osteomyelitis and perform debridement when necessary. These additional data and treatments did not appear to alter short-term outcomes including reoperation, readmission, and time to discharge in our subgroup. It is possible that they influenced antibiotic duration, but this was beyond the scope of this study.

The decision was made to exclude patients without positive joint aspiration or elevated temperature, ESR, CRP, or WBC values to capture higher-acuity patients which would warrant elevated concern for possible septic joints. We selected these variables as they reflect components of Kocher’s criteria for diagnosing a septic hip as well as their later modification with the addition of CRP [12,24]. Surgeons would likely have less hesitation to obtain a preoperative MRI when these values are normal. Furthermore, requiring patients in the comparison group to have had at least one pre- or postoperative MRI via our methodology resulted in the exclusion of patients without any preoperative or postoperative MRI. This decision was made so that concurrent osteomyelitis could be diagnosed if present with septic arthritis. The excluded cohort likely had similar or better outcomes than those included in this study as an MRI would have been less likely obtained had the patient improved after surgery. Similarly, our population did not include patients that were diagnosed with another pathology that did not require surgery such as transient synovitis or cellulitis. Although we recognize that identifying these pathologies is an additional benefit of preoperative MRI, it was beyond the scope of this study.

Further research is warranted to investigate medium- to long-term outcomes when delay is necessary to obtain preoperative MRI. Additional studies could investigate AVN or arthritis rates in patients that had MRI prior to debridement. Likewise, it would be beneficial to develop new tools to decrease the time to obtain preoperative MRI. Several academic centers are developing rapid MRI sequence protocols that show promise in decreasing the need for anesthesia and time to surgery [25]. Our data suggest that patient outcomes are not adversely affected by obtaining preoperative MRI despite the delay in time to OR. Although we did not demonstrate any differences in reoperation, readmission, or time to discharge, 40.4% of patients with septic arthritis were found to have concurrent osteomyelitis, which may have influenced the extent of debridement and antibiotic duration. We believe this study provides sufficient evidence of non-inferior outcomes when obtaining preoperative MRI in patients with septic arthritis and/or osteomyelitis to support a randomized controlled trial to investigate this topic. Although such a study would prove most valuable to providers managing pediatric patients with atraumatic extremity pain, the decision to obtain MRI and timing of surgery should be left to the discretion of the treating surgeon.

## Figures and Tables

**Table 1:** A comparison of patients with and without preoperative MRI with mean/median [IQR values] provided for continuous data and raw values and percentages provided for categorical data.

	No Preop MRI (N = 29)	Preop MRI (N = 75)	Overall (N = 104)	*p*-Value
Demographics				
Age (years)	6.2/7.0 [1.0, 10.0]	7.6/6.0 [3.0, 12.0]	7.3/6.0 [3.0, 11.0]	0.17 _I_
Female	14 (48.3%)	26 (34.7%)	40 (38.5%)	0.20 _II_
Presentation				
Symptom Duration (Days)	6.6/4.0 [2.0, 7.0]	7.5/5.0 [3.0, 12.0]	7.3/5.0 [2.5, 7.0]	0.58 _III_
Temperature (°C)	37.8/37.6 [37.2, 38.3]	37.5/37.4 [36.9, 38.1]	37.6/37.5 [36.9, 38.2]	0.18 _I_
ESR (mm/h)	49.5/44.5 [30.0, 89.5]	52.3/59.0 [37.0, 75.0]	51.8/58.0 [32.0, 80.0]	0.90 _I_
CRP (mg/dL)	10.9/7.6 [3.2, 12.7]	11.6/9.0 [4.1, 14.4]	11.5/8.8 [3.5, 14.4]	0.39 _III_
WBC (K/µL)	16.7/14.0 [10.0, 18.9]	14.2/13.8 [9.1, 16.7]	14.8/13.9 [9.2, 17.6]	0.31 _III_
Positive Blood Culture	8 (27.6%)	23 (30.7%)	31 (29.8%)	0.79 _II_
Preoperative Joint Aspiration	21 (72.4%)	21 (28.0%)	42 (40.4%)	<0.0001 _II_
Positive Joint Aspiration	18/21 (85.7%)	14/21 (67.7%)	32/42 (76.2%)	0.15 _II_
Timing of OR				
Same Day as Admission	13 (44.8%)	15 (20.0%)	28 (26.9%)	0.0037 _II_
Day after Admission	14 (48.3%)	33 (44.0%)	47 (45.2%)	-
2 or More Days after MRI	2 (6.9%)	27 (36.0%	29 (27.9%)	-
Final Diagnosis				
Osteomyelitis	4 (13.8%)	43 (57.3%)	47 (45.2%)	<0.0001 _II_
Septic Joint	19 (65.5%)	15 (20.0%)	34 (32.7%)	-
Septic Joint + Osteomyelitis	6 (20.7%)	17 (22.7%)	23 (22.1%)	-

I: Unequal variance two sample *t*-test; II: Chi-Square *p*-value; III: Wilcoxon rank sum *p*-value.

**Table 2:** Outcome data for the overall cohort comparing patients with and without preoperative MRI with mean/median [IQR] values provided for continuous data and raw values and percentages provided for categorical data.

	No Preop MRI (N = 29)	Preop MRI (N = 75)	Overall (N = 104)	*p*-Value
Outcomes				
Time from OR to Discharge (Days)	5.8/5.0 [4.0, 8.0]	5.4/4.0 [3.0, 7.0]	5.5/5.0 [3.0, 7.0]	0.57 _II_
Total Number of Debridements				
1	21 (72.4%)	56 (74.7%)	77 (74.0%)	0.23 _III_
2	7 (24.1%)	10 (13.3%)	17 (16.3%)	-
3+	1 (3.4%)	9 (12.0%)	10 (9.6%)	-
Total Admissions				
1	26 (89.7%)	60 (80.0%)	86 (82.7%)	0.24 _I_
2+	3 (10.3%)	15 (20.0%)	18 (17.3%)	-
Total MRIs				
1	23 (79.3%)	51 (68.0%)	74 (71.2%)	0.23 _III_
2	5 (17.2%)	12 (16.0%)	17 (16.3%)	-
3+	1 (3.4%)	12 (16.0%)	13 (12.5%)	-

I: Chi-Square *p*-value; II: Wilcoxon rank sum *p*-value; III: Fisher Exact *p*-value.

**Table 3:** A comparison of patients found to have septic arthritis with and without preoperative MRI with mean/median [IQR] values provided for continuous data and percentages provided for categorical data.

	No Preop MRI (N = 25)	Preop MRI (N = 32)	Overall (N = 57)	*p*-Value
Demographics				
Age (years)	6.4/6.0 [3.0, 10.0]	5.8/3.5 [2.0, 11.0]	6.1/5.0 [2.0, 11.0]	0.72 _II_
Female	11 (44%)	11 (34.4%)	22 (38.6%)	0.46 _I_
Presentation				
Symptom Duration (Days)	5.0/6.4 [2.0, 7.0]	6.8/4.5 [2.5, 7.0]	6.1/5.0 [2.0, 7.0]	0.71 _II_
Temperature (°C)	37.8/37.6 [37.4, 38.3]	37.6/37.5 [37.2, 38.0]	37.7/37.6 [37.2, 38.1]	0.27 _II_
ESR (mm/h)	50.0/60.5 [31.0, 91.0]	58.3/58.0 [31.0, 80.0]	55.8/58.0 [31.0, 90.0]	0.57 _II_
CRP (mg/dL)	12.5/8.3 [4.0, 16.7]	9.4/8.8 [4.7, 10.6]	10.3/8.7 [4.1, 12.4]	0.85 _II_
WBC (K/µL)	15.5/12.8 [10.0, 18.0]	14.3/14.4 [10.3, 15.9]	14.8/14.0 [10.0, 17.0]	0.58 _II_
Positive Blood Culture	7 (28.0%)	9 (28.1%)	16 (28.1%)	0.77 _III_
Preoperative Joint Aspiration	20 (80.0%)	15 (46.9%)	35 (61.4%)	0.01 _I_
Positive Joint Aspiration	18/20 (92.0%)	11/15 (73.3%)	29/35 (50.9%)	0.20 _I_
Timing of OR				
Same Day as Admission	12 (48.0%)	7 (21.9%)	19 (33.3%)	0.023 _I_
Day after Admission	12 (48.0%)	16 (50.0%)	28 (49.1%)	-
2 or More Days after MRI	1 (4.0%)	9 (28.1)%	10 (17.5%)	-

I: Chi-Square *p*-value; II: Wilcoxon rank sum *p*-value; III: Fisher Exact *p*-value.

**Table 4:** Outcome data for the septic arthritis cohort comparing patients with and without preoperative MRI with mean/median [IQR] values provided for continuous data and raw values and percentages provided for categorical data.

	No Preop MRI (N = 25)	Preop MRI (N = 32)	Overall (N = 57)	*p*-Value
Outcomes				
Time from OR to Discharge (Days)	6.7/5.0 [4.0, 8.0]	6.2/5.0 [4.0, 7.5]	6.4/5.0 [4.0, 8.0]	0.77 _I_
Total Number of Debridements				
1	17 (68.0%)	27 (84.4%)	44 (77.2%)	0.17 _II_
2	7 (28.0%)	3 (9.4%)	10 (17.5%)	-
3+	1 (4.0%)	2 (6.3%)	3 (5.3%)	-
Total Admissions				
1	22 (88.0%)	27 (84.4%)	49 (86.0%)	1 _II_
2+	3 (12.0%)	5 (15.6%)	8 (14.0%)	-
Total MRIs				
1	20 (80.0%)	22 (68.8%)	42 (73.7%)	0.46 _II_
2	4 (16.0%)	5 (15.6%)	9 (15.8%)	-
3+	1 (4.0%)	5 (15.6%)	6 (10.5%)	-

I: Wilcoxon rank sum *p*-value; II: Fisher Exact *p*-value.

**Table 5:** Rates of concurrent osteomyelitis (OM) and bony debridement of the OM at the time of surgery in patients with septic arthritis comparing those with and without preoperative MRI.

Joint	Preop MRI	No Preop MRI	Total	*p*-Value
Hip	13	9	22	
Concurrent OM	8	2	10	0.068 _I_
OM Debridement	3	0	3	0.121 _I_
Knee	8	8	16	
Concurrent OM	4	4	4	1.000 _I_
OM Debridement	0	0	0	1.000 _I_
Shoulder	5	3	8	
Concurrent OM	3	1	4	0.465 _I_
OM Debridement	1	0	1	0.408 _I_
Elbow	3	2	5	
Concurrent OM	1	1	2	0.709 _I_
OM Debridement	1	1	2	0.709 _I_
Ankle	3	1	4	
Concurrent OM	3	0	3	0.046 _I_
OM Debridement	3	0	3	0.046 _I_
Wrist	0	2	2	
Concurrent OM	0	0	0	1.000 _I_
OM Debridement	0	0	0	1.000 _I_
Total	32	25	57	
Concurrent OM	17	6	23	0.026 _I_
OM Debridement	8	1	9	0.031 _I_

I: Chi-Square *p*-value.

## Data Availability

The data presented in this study are available on request from the corresponding author. The data are not publicly available due to privacy and ethical concerns.
